# Pulmonary embolism in neurocritical care-introduction of a novel grading system for risk stratification: the Frankfurt AMBOS score

**DOI:** 10.1007/s10143-020-01310-6

**Published:** 2020-05-12

**Authors:** Daniel Dubinski, Fee Keil, Sae-Yeon Won, Bedjan Behmanesh, Kolja Jahnke, Volker Seifert, Christof Geisen, Juergen Konczalla, Christian Senft

**Affiliations:** 1 Department of Neurosurgery, University Hospital, Goethe University, Frankfurt, Germany; 2 Johann Wolfgang Goethe-Universität, Frankfurt am Main, Frankfurt, Germany; 3 Institute of Neuroradiology, Goethe University, Frankfurt, Germany; 4 Department of Neurology, University Hospital, Goethe University, Frankfurt, Germany; 5 Institute for Transfusion Medicine and Immunohematology, Goethe University, Frankfurt, Germany

**Keywords:** Blood group, Neurocritical care, Deep vein thrombosis, Thromboembolism, Risk factor

## Abstract

Pulmonary embolism (PE) due to deep vein thrombosis is a complication with severe morbidity and mortality rates. Neurocritical care patients constitute an inhomogeneous cohort with often strict contraindications to conventional embolism treatment. The aim of the present study is to identify risk factors for pulmonary embolism for intensified risk stratification in this demanding cohort. In this retrospective analysis, 387 neurocritical care patients received computed tomography for clinical suspicion of PE (304 neurosurgical and 83 neurological patients). Analysed parameters included age, gender, disease pattern, the presence of deep vein thrombosis, resuscitation, in-hospital mortality, present anticoagulation, coronary artery disease, diabetes mellitus, smoking status, hypertension and ABO blood type. Computed tomography confirmed 165 cases of pulmonary embolism among 387 patients with clinical suspicion of pulmonary embolism (42%). Younger age (*p* < 0.0001), female gender (*p* < 0.006), neurooncological disease (*p* < 0.002), non-O blood type (*p* < 0.002) and the absence of Marcumar therapy (*p* < 0.003) were identified as significant risk factors for pulmonary embolism. On the basis of the identified risk factors, the AMBOS score system is introduced. Neurocritical care patients with high AMBOS score are at elevated risk for PE and should therefore be put under intensified monitoring for cardiovascular events in neurocritical care units.

## Introduction

Acute pulmonary embolism (PE) from deep vein thrombosis (DVT) resulting in venous thromboembolism (VTE) is a life-threatening event that requires intensive care treatment with urgent pharmacological and/or mechanical intervention. PE can present asymptomatically, with unspecific symptoms such as mild dyspnoea, chest pain, anxiety or in its fulminant form with cardiac arrest due to acute heart failure [7].

The current gold standard in VTE treatment consists of pharmacological anticoagulation [18] [15]. Conventional anticoagulation with low-molecular-weight heparin (LMWH) followed by vitamin K antagonists is nowadays superseded by direct oral anticoagulants (DOACs) [11, 20]. In PE patients with persistent hypotension and shock, interventional thrombolysis through catheter-based local infusion of thrombolytic agents into the pulmonary artery or the percutaneous thrombus fragmentation is seen as the ultima ratio [13, 14]. However, these treatment modalities are often strictly contraindicated in neurocritical care patients. Therefore, identifying patients at high risk for PE is of crucial importance for the neurointensivist. Established PE risk factors can be separated into inherited risk factors such as haemostaseologic defects (factor V Leiden; Protein C, S deficiency etc.) [17] and acquired risk factors such as bed rest, major trauma, major surgery, malignancy and varicose veins [19]. In the neurointensive care setting, patients often exhibit combined relative contraindications for anticoagulation such as acute intracranial haemorrhage, severe uncontrolled hypertension, TBI, history of recent stroke, thrombocytopenia or intracranial surgery, thus limiting therapeutic options [1, 9]. However, a statement for healthcare professionals from the Neurocritical Care Society in 2016 recommended the initiation of chemical VTE prophylaxis with s.c. LMWH in patients with stable intracranial hematomas within 48 h of hospital admission [18]. Previously, several scoring systems have been introduced in outpatient cohorts and general large patient cohorts for PE risk stratification such as the modified Wells criteria and the revised Geneva score [12, 22]. However, because of the variable presentation of PE, the limited therapeutic options and the great impact on the posttreatment opportunities, proper risk stratification has direct clinical implication in order to establish prophylactic as well as therapeutic treatment strategies for the neurocritical ill.

## Materials and methods

### Patients and data collection

The present study was approved by the clinical ethics committee of the University of Frankfurt (20-683). All patients over 18 years old who were treated at our neurocritical intensive care unit (ICU) from 2010 to 2017 with clinical suspicion of PE during their stay were identified retrospectively using the electronic database. Clinical suspicion and therefore indication for thoracic CT scan were the acute onset of one or the combination of the following symptoms: collapse upon mobilization, shock, hypotonia, tachycardia, dyspnoea, chest pain or dip in oxygen saturation. Exclusion criteria were pre-existing haematological disorders and the lack of knowledge on ABO blood type. Parameters investigated using patients’ medical records were the presence of deep vein thrombosis, resuscitation, in-hospital mortality, ABO blood type, present anticoagulation, coronary artery disease, diabetes mellitus and hypertension. For VTE prophylaxis, all patients received s.c. LMWH within 48 h of admission. If the patient underwent neurosurgery, half dosage of prophylactic LMWH was administered 10 h postoperative and regular LMWH prophylaxis initiated on the first postoperative day. In patients with elective surgical procedures, phenprocoumon therapy was paused at least 2 weeks prior surgery and switched to LMWH, which was paused on the day of surgery. However, if patients presented with acute haemorrhage, immediate reversion was started with weight and inter norm ratio (INR) adapted prothrombin complex concentrate (PCC) and 10 mg phytomenadione/day (Konakion®) was initiated.

### Study design

The present analysis is a retrospective, single-centre observational study of neurocritical care patients. The aims of the study were (1) to observe the incidence of clinical suspicion and PE verification, (2) examine the leading risk factors and (3) establish a novel risk stratification grading system for neurocritical care patients.

### Statistics

Data analysis was performed with BiAS (Version 11.06.2017). For parametric parameters, the ANOVA test was used. For nonparametric parameters, the Wilcoxon-Mann-Whitney test was used. To assess the impact of the variables, odds ratio (OR) with 95% confidence intervals (CI) were calculated. Results with *p* ≤ 0.05 were considered statistically relevant.

## Results

### Demographics and clinical characteristics

**Fig. 1:**
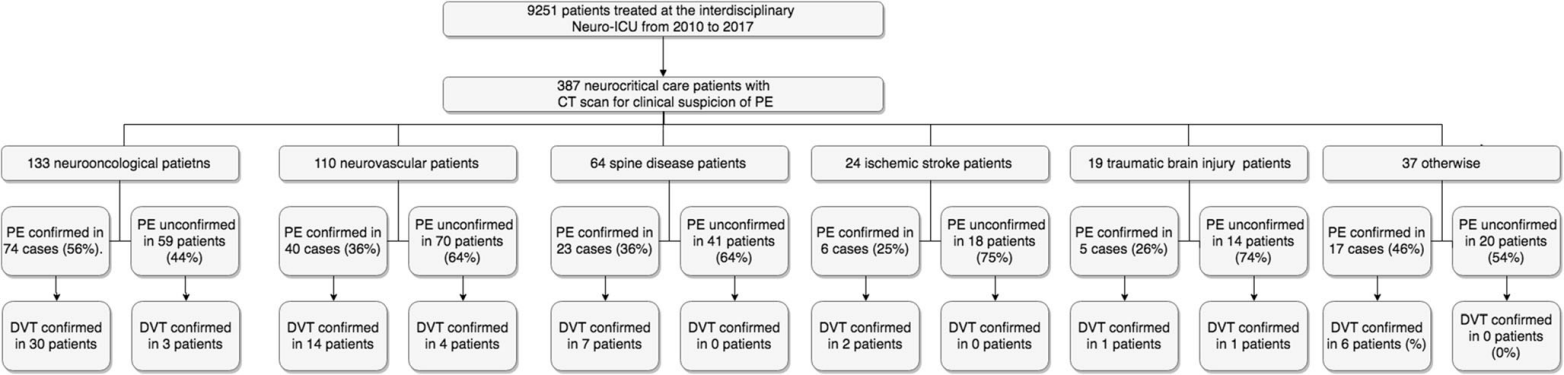
Flow diagram displaying patient allocation

Between January 2010 and December 2017, 9251 patients were admitted to our interdisciplinary Neuro-ICU. A total of 387 neurocritical care patients underwent diagnostic imaging (thoracic CT scan) due to clinical suspicion of PE at the authors’ institution. Our Neurocritical Care Unit is of interdisciplinary character with neurological and neurosurgical patients. Patient characteristics included 191 female cases (49%) with median age of 71 years (SD 14 years). PE was radiologically confirmed in 165 out of 9251 patients treated in the abovementioned time frame (1.8%). The largest cohort undergoing CT scan for clinical suspicion of PE were 133 neurooncological NICU patients with positive PE confirmation in 74 cases (55%). A total of 110 NICU patients with neurovascular disease underwent CT scan for PE suspicion, with PE confirmation in 40 cases (36%). A total of 64 patients with primary spine disease undergoing CT scan analysis had a radiological confirmation in 23 cases (35%). There were 24 stroke patients with PE proof in 6 patients (25%). A total of 19 patients with traumatic brain injury were identified with radiological CT scan for PE, which were confirmed in 5 cases (26%). There were 37 patients with other neurocritical care conditions analysed for clinical suspicion of PE; a radiological confirmation was obtained in 17 cases (46%). Disease allocation of PE cohort is displayed in Fig. 1. Overall, CT scan confirmation of PE was obtained in 165 cases (91 female; 55%) with 51 cases (30%) of central- and 114 cases of periphery pulmonary embolism (70%). PE patients had a median age of 68 (SD 13 years). Among the 165 PE patients, 62 patients had ultrasound confirmation of DVT (37%), whereas among the 222 PE negative patients had 8 cases of DVT (4%). Median PE occurrence was on day 7 (SD 10 days). Among the PE cases, 52 patients (32%) had had surgery with median PE verification on the 6th day postoperatively (range 6 days). In the 113 nonsurgical patients (68%), PE was diagnosed on median day 7 (range 11 days) after admission. Resuscitation was performed in 16 of the 165 PE cases and 10 in the PE negative cohort. Patient characteristics are displayed in Table 1.

**Table 1:** Characteristics of neurocritical care patients who received thoracic CT scan for clinical suspicion of pulmonary embolism. CT: computed tomography; PE: pulmonary embolism; DVT: deep vein thrombosis; NOACs: novel oral anticoagulants; SD: standard deviation

Patients characteristics	*n* = 387
Age, mean (SD)	71 (14)
Sex (female), *n* (%)	191 (49)
CT scan confirmation of PE, mean (%)	165 (55)
Central PE, *n* (%)	51 (30)
Peripheral PE, *n* (%)	114 (70)
Ultrasound confirmed DVT, *n* (%)	70 (18)
Neurosurgical disease, *n* (%)	304 (79)
Neurological disease, *n* (%)	83 (21)
Neurooncology (PE confirmed) *n* (%)	74 (45)
Neurovascular (PE confirmed) *n* (%)	40 (24)
Spine disease (PE confirmed) *n* (%)	23 (14)
Stroke (PE confirmed) *n* (%)	6 (4)
Traumatic brain injury (PE confirmed) *n* (%)	5 (3)
Otherwise (PE confirmed) *n* (%)	6 (16)
Median PE verification, day (SD)	6.5 (8.5)
Resuscitation, *n* (%)	26 (7)
In hospital mortality, *n* (%)	24 (6)
Blood type O, *n* (%)	144 (37)
Blood type A, *n* (%)	172 (44)
Blood type B, *n* (%)	50 (13)
Blood type AB, *n* (%)	22 (6)
Rhesus factor, *n* (%)	325 (84)
Smoker, *n* (%)	70 (18)
Hypertension, *n* (%)	203 (52)
Coronary artery disease, *n* (%)	95 (25)
Diabetes mellitus, *n* (%)	80 (21)
Anticoagulation, *n* (%)	110 (28)
NOACs, *n* (%)	19 (5)
Aspirin, *n* (%)	73 (19)
Marcumar, *n* (%)	18 (5)

### Inherited risk factors for pulmonary embolism in neurocritical care patients

Among the analysed cohort patients with CT scan confirmation of PE, the mean age was 68 years (SD 13) opposite to patients without PE, which had a mean age 75 years (SD 14). Therefore, patients’ age was identified as an inherited and highly significant risk factor for PE in neurocritical care, *p* < 0.0001 (Students *t*) (Table 2).

**Table 2:** Cohort allocation according to CT scan verified pulmonary embolism vs. no pulmonary embolism verification: CT: computed tomography; PE: pulmonary embolism; DVT: deep vein thrombosis; NOACs: novel oral anticoagulants; SD: standard deviation

Patients characteristics (*N* = 387)	Pulmonary embolism		
	Yes (*N* = 165)	No (*N* = 222)	*p* value
Age, mean (SD)	68 (41)	75 (34)	*0.0001*
Sex (female), *n* (%)	90 (55)	101 (44)	0.04
Central PE, *n* (%)	51 (31)	–	
Peripheral PE, *n* (%)	114 (69)	–	
Ultrasound confirmed DVT, *n* (%)	65 (37)	8 (4)	*0.0001*
Median PE verification, day (SD)	6 (6)	–	
Resuscitation, *n* (%)	16 (10)	10 (3)	0.06
In hospital mortality, *n* (%)	10 (6)	14 (6)	n.s
Blood type O, *n* (%)	48 (29)	96 (43)	*0.005*
Blood type A, *n* (%)	74 (45)	98 (44)	n.s
Blood type B, *n* (%)	30 (18)	20 (8)	*0.009*
Blood type AB, *n* (%)	13 (8)	10 (5)	n.s.
Rhesus factor, *n* (%)	138 (84)	187 (84)	n.s.
Smoker, *n* (%)	28 (17)	42 (19)	n.s.
Hypertension, *n* (%)	78 (47)	125 (56)	0.08
Coronary heart disease, *n* (%)	32 (19)	63 (28)	*0.04*
Diabetes mellitus, *n* (%)	27 (16)	51 (23)	n.s.
Anticoagulation, *n* (%)	36 (22)	74 (33)	*0.01*
NOACs, *n* (%)	10 (6)	9 (4)	n.s.
Aspirin, *n* (%)	24 (15)	49 (22)	0.06
Marcumar, *n* (%)	2 (1)	16 (7)	*0.007*

Significant *p* values are highlighted in italics

Furthermore, patients with PE had significantly less often blood type O (29%) and statistically significant higher presence of non-O blood type (71%) in general and blood type B (18%) in specific, (*p* = 0.005 and *p* = 0.009, respectively, Chi-square test). Neurocritical care patients with non-O blood type were therefore identified with an inherited elevation of PE risk. The presence of Rhesus factor however was not associated with an elevated risk for PE. Furthermore, patients’ sex was significantly associated with PE risk: The presence of female patients with PE was observed (55% of females in the PE cohort vs. 44% in the PE negative group, *p* < 0.006).

### Acquired risk factors for pulmonary embolism in neurocritical care patients

The presence of a neurooncological disease pattern was highly associated with PE risk. PE was confirmed in 56% of the neurooncological cohort vs. 41% in the non-neurooncological cohort (*p* < 0.0002).

Sonographic confirmation of DVT was obtained in 62 cases in the PE group vs. 8 cases in the PE negative group (*p* < 0.00001; OR: 16.25, 95% CI: 7.50; 35.20) concluding that DVT is a strong risk factor for PE, although the analysis was performed after clinical manifestation and CT scan investigation. The post incidence confirmation of DVT is not inconceivable for DVT post PE and therefore cannot be viewed as a direct risk factor.

Analysed parameters that were not significantly associated with PE in neurocritical care patients include the presence of diabetes mellitus, hypertension and smoking status.

### Secondary effects of pulmonary embolism in neurocritical care patients

The presence of PE showed no significant difference in terms of in hospital mortality with 10 cases (6%) of PE confirmed group vs. 14 cases (6%) in the PE negative cohort. The necessity of resuscitation (defibrillation, chest compression, intubation and pharmacological intervention) after clinical manifestation of PE did not show statistical significance between the analysed groups. However, a tendency of higher resuscitation rates among the PE confirmed group was observed (16 cases (10%) in the PE confirmed group vs. 10 cases (3%) in the PE negative group, *p* > 0.05).

### Protective factors against pulmonary embolism

The general presence of anticoagulation therapy at admission showed a protective result in terms of PE manifestation: 36 patients (22%) with PE confirmation had anticoagulation therapy vs. 74 patients (33%) of PE negative group with anticoagulation therapy (*p* < 0.01). Subgroup analysis revealed that neurocritical patients with phenprocoumon therapy (Marcumar®;Warfarin®) were significantly less affected by PE. In the PE negative cohort, 16 patients were on phenprocoumon therapy (7%) vs. 2 (1%) in the PE confirmed subgroup (*p* < 0.007). A borderline significant protective effect of aspirin® was further observed: 49 patients (22%) in the PE negative group vs. 24 (15%) in the PE confirmed subgroup (*p* < 0.06).

Contra-intuitively, our analysis revealed the presence of coronary artery disease (CAD) as protective against PE: 63 patients (28) with history of CAD had no PE vs. 32 (19%) of PE confirmed cases with CAD history (*p* < 0.04).

### The Frankfurt AMBOS score for risk stratification of pulmonary embolism

**Fig. 2:**
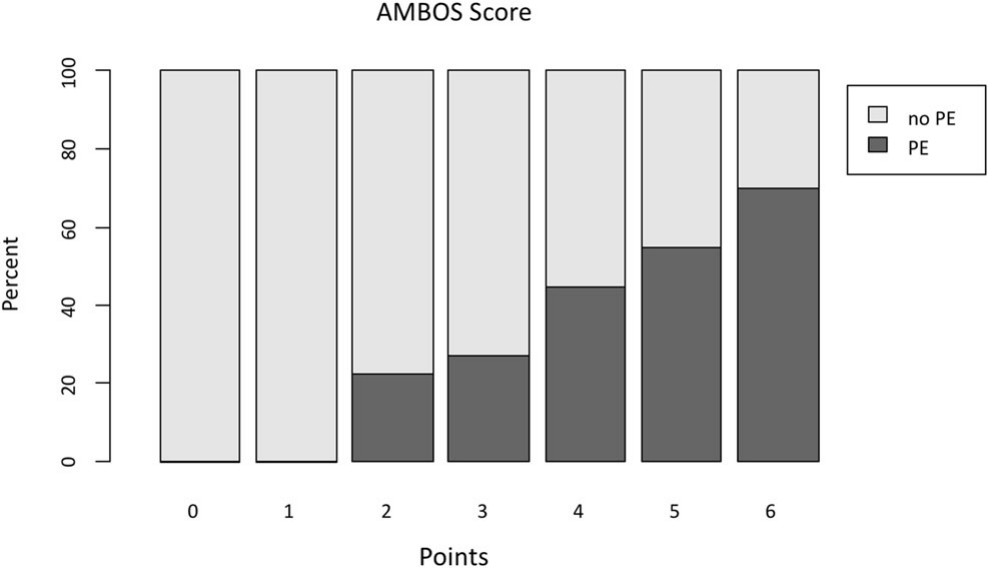
The Frankfurt AMBOS score. Y axis showing the percentage of analysed patients, the X axis displays the AMBOS points. Higher AMBOS points are associated with elevated risk for PE

In univariate and multivariate analysis, the following characteristics were statistically significant risk factors for PE in neurocritical care patients: Age (*p* < 0.0001), non-O blood type (*p* = 0.005), (neuro-)oncological disease (*p* < 0.0002) and female sex (*p* < 0.04) (Table 3). The only significant protective factor against PE was the Marcumar intake before or at admission (*p* < 0.007). See Table 4. We therefore developed the Frankfurt AMBOS score for PE risk stratification among all neurocritical patients with clinical suspicion of PE: in our cohort (*n* = 387), 0% of all patients with 0 points had an PE as was the case for patients with 1 point. A total of 22% of the patients with 2 points (*n* = 8) had PE. Patients with 3 points (*n* = 23/27%) had PE and 4 points (*n* = 59/44%) of the analysed cohort. A total of 52 patients (54%) undergoing CT scan for clinical suspicion of PE with 5 points had verified PE manifestation. A total of 6 points were observed in 21 patients (70%) with confirmed PE. Patients with 6 points (*n* = 21/70%) had CT scan confirmed PE. See Fig. 2.

**Table 3:** The Frankfurt AMBOS score for risk stratification of PE in neurocritical care

Variable	Points
Age: < 69	1
Marcumar therapy: no	2
Blood type: non-O	1
Oncological disease: yes	1
Sex: female	1

**Table 4:** Uni- and multivariate analysis of included patients into the AMBOS score. SD: standard deviation

Patients characteristics (*N* = 387)	Pulmonary embolism		Univariate			Multivariate		
	Yes (*N* = 165)	No (*N* = 222)	*p* value	OR	95% CI	*p* value	OR	95% CI
Age, mean (SD)	68 (41)	75 (34)	0.0001	1.5	4.25–9.75	0.006	1.84	1.19–2.84
Sex (female), *n* (%)	90 (55)	10 (44)	0.006	1.5	1.13–2.56	0.004	0.64	0.41–0.97
Neurooncology vs. non-neurooncology *n*, (%)	74 (56)	91 (41)	0.002	2.2	1.46–3.44	0.016	1.69	1.10–2.67
Blood type O vs. non-O, *n* (%)	48 (29)	96 (43)	0.002	1.8	1.21–2.84	0.003	0.50	0.32–0.79
Marcumar vs. no Marcumar, *n* (%)	2 (1)	16 (7)	0.003	6.3	1.43–27.92	0.043	0.26	0.07–0.96

## Discussion

The present study analyses inherited and acquired risk factors for presence of PE in neurocritical care patients in whom there is a clinical suspicion of PE. The identified risk factors are included in the Frankfurt AMBOS risk stratification score. The new, proposed score can simplify the identification of neurocritically ill patients at risk for PE in the clinical setting.

The identified risk factors of younger age and female sex are intriguing since the revised Geneva score describes older age to be a risk factor for PE [10]. A possible explanation could be (at least for patients with intracranial haemorrhage) the hypercoagulability state and an increased haemostaseologic response in younger patients upon intracranial haemorrhage. [6] The protective nature of anticoagulation therapy in general and vitamin K antagonism in specific appears to be consequent taking the duration of quick normalization into consideration. The thrombogenic effects of oncological and, as in our investigation, neurooncological disease as a risk factor for VTE are also coherent with the scientific literature [16, 21]. The critical effect of ABO antigens present on red blood cells and endothelium led to the identification of patients’ blood type as a risk factor for postoperative bleeding and poor outcome in several neurosurgical diseases [3, 4]. The elevated plasma levels of von Willebrand factor (vWF) and factor VIII (F-VIII) in non-O blood type patients contribute to a significant elevation of PE risk in neurocritical care.

Hereinafter, our study adds additional information for future development of treatment regimes in neurointensive care patients with PE. Taken together, the majority of scientific literature focuses on general patient cohort without intracranial affection. Therefore, proper risk stratification is urgently needed to establish prophylactic and treatment protocols for PE in neurocritical care. The Frankfurt AMBOS score provides a risk stratification based on a large cohort of neurocritically ill patients and adds important information for future treatment consensus. At present, a coherent treatment recommendation in neurocritical care patients with fulminant embolism is absent. However, several case reports describe successful systemic thrombolysis to be effective despite present contraindications [2, 5, 8].

Our study has several strengths. We did analyse a sufficient number of patients and therefore reach a sufficient statistical power to verify or findings. However, missing data on patients’ blood type excluded 18 patients, but the blood type distribution in our cohort remained equal to the normal German distribution, which strengthens the representability of our cohort.

A weakness of our study is the fact that we did analyse only the cases with clinical suspicion of PE and could therefore have missed (fatal) PE cases that were not identified (and treated) as such. However, this is a single-centre study and is of retrospective character, which could compromise a bias itself. Furthermore, the exact evaluation of VKA reversal is lacking since it was (at least in part) initiated prior to admission.

In conclusion, the Frankfurt AMBOS grading score can help neurointensivists with a quick and reliable risk stratification in clinical suspected PE. The score adds to the discussion of intensified monitoring and prolonged prophylactic LWMH treatment in this demanding patient cohort.
